# Primary Cerebral Venous Thrombosis in a Patient with Immune Thrombocytopenic Purpura

**DOI:** 10.1155/2022/1346269

**Published:** 2022-08-19

**Authors:** M. Taher Farfouti, Christina Masri, Mike Ghabally, George Roumieh

**Affiliations:** ^1^MRCP (UK), FRCP (London), Department of Neurology, Aleppo University, Faculty of Medicin, Aleppo, Syria; ^2^University of Aleppo, Department of Internal Medicine, Faculty of Medicine, Aleppo, Syria; ^3^University of Aleppo, Department of Internal Medicine, Division of Cardiology, Faculty of Medicine, Aleppo, Syria; ^4^University of Aleppo, Department of Internal Medicine, Division of Gastroenterology, Aleppo, Syria

## Abstract

**Introduction:**

Immune thrombocytopenic purpura is an autoimmune hematological disorder characterized by low platelet level due to its destruction through autoimmune antibodies. Cerebral venous thrombosis is a serious condition defined by a thrombosis in the cerebral venous sinuses that occurs mostly in the presence of a hypercoagulable state. Hemorrhage and thrombosis are processes with a paradoxical etiology; thus, the association between these two conditions seems to be extremely rare. *Case Presentation*. We herein report a case of a 19-year-old female with a chief compliant of generalized tonic-clonic episode, severe headache, and blurred vision. Physical examination was significant for a bilateral Babinski's sign and severe bilateral papilledema. Laboratory workup, computed tomography, and magnetic resonance imaging were normal except for severe thrombocytopenia. Magnetic resonance venography was diagnostic for cerebral venous thrombosis. Her past medical history was significant for immune thrombocytopenic purpura that was treated with prednisolone 40 mg per day which posed a therapeutic challenge. High-dose prednisolone and platelet transfusion were initiated; enoxaparin was administrated and switched to warfarin after stabilization of platelet count. The patient was neurologically intact after 14 days of appropriate treatment and was on follow-up. Many hypotheses were suggested to explain the unexpected thrombotic events in a patient with immune thrombocytopenic purpura which were related to the disease etiology or treatment, taking into account common risk factors (such as age, obesity, smoking, hypertension, diabetes mellitus, dyslipidemia, splenectomy, and oral contraceptive agents).

**Conclusion:**

The association between immune thrombocytopenic purpura (which is a major risk factor for bleeding) and cerebral venous thrombosis ( which is caused by a thromboembolic event )has carried a major challenge to the management plan. We believe that immune thrombocytopenic purpura and its treatment methods should be taken into consideration as risk factors for thromboembolic phenomenon.

## 1. Background

Immune thrombocytopenic purpura (ITP) is an acquired autoimmune hematological disease. It is characterized by antibody-mediated platelet destruction causing thrombocytopenia [1, 2].

The clinical manifestations are widely diversified, and some patients can be completely asymptomatic or express some minor bleeding symptoms including purpura, petechiae, epistaxis, and hemorrhage tendency. On the other hand, it may present with lethal bleeding such as intracranial hemorrhage, especially during severe thrombocytopenia [1, 2].

=The management plan in each condition is varied ,depending onthe manifestations. Asymptomatic patients may require only close observation. However, the main agents used in the treatment of ITP include corticosteroids, intravenous immunoglobulin (IVIG), thrombopoietin receptor agonist, and splenectomy [1].

Cerebral venous thrombosis (CVT) is a relatively uncommon but serious condition that occurs mostly in the presence of a hypercoagulable state, which has several causes including pregnancy, inherited and acquired thrombophilia, infections, and malignancies [1].

Clinical manifestations include confusion, altered mental status, focal or generalized seizures, focal neurological deficits, and signs of increased intracranial pressure (ICP) such as severe headache and papilledema [1].

The mainstay of the management of CVT is anticoagulation, as well as the management of the underlying cause.

According to the paradoxical etiology for these two conditions, its association seems to be extremely rare; however, in our study, we present a case of a young female who developed these two disorders simultaneously.

## 2. Case Presentation

A 19-year-old Caucasian female was presented to the emergency department with a chief complaint of two-day history of severe bilateral non-pulsating headache and blurred vision. The patient experienced a generalized tonic-clonic episode in our emergency department; thus, diazepam 10 mg IV was given twice followed by 15 mg/kg phenytoin. Her seizures were controlled and she was admitted to the intensive care unit.

Past medical history was significant for recurrent episodes of epistaxis and purpura that started a year before the current presentation. Routine workups revealed the presence of severe thrombocytopenia while other lab tests (including thyroid function tests, liver function tests, renal function tests, electrolytes, CRP, LDH, PT, INR, PTTK, fibrinogen, RF, ANA, anti-dsDNA, antiphospholipid Ab IgM and IgG, anticardiolipin, FOB, and urinalysis) were within normal limits. HBV, HCV, and HIV screening was also negative. A blood smear revealed isolated thrombocytopenia. Bone marrow biopsy revealed an increase in the size and count of megakaryocytes. Past surgical, family, and social history was unremarkable. She is sexually inactive and does not use oral contraceptive pills or any other medications. Unfortunately, antiplatelet antibody tests are unavailable in our county. Therefore, the patient was diagnosed with ITP by ruling out other possible causes. Prednisolone 40 mg PO was started and continued regularly until presentation. On follow-up, the patient demonstrated fluctuating response. However, symptoms have recurred.

Physical and neurological exams were all normal except for a positive bilateral Babinski's sign. There were no focal signs.

**Figure 1:**
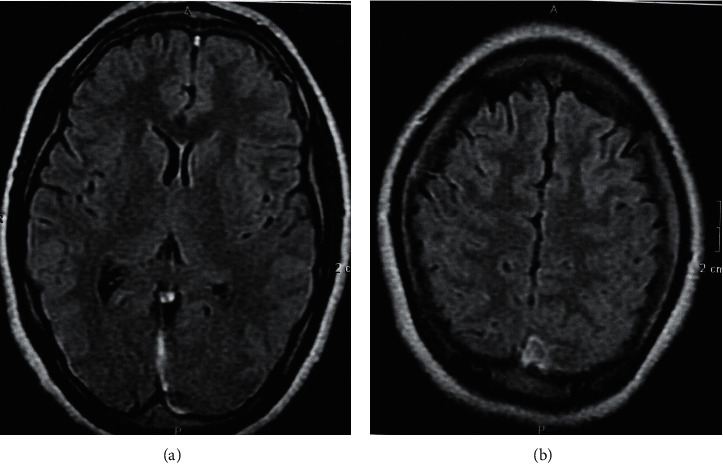
Brain MRV scan. (a) Almost complete absence of the intensity (supra-tentorial) in the left transverse, and the occipital venous sinuses. (b) Almost complete absence of the intense in the sagittal venous sinus.

Laboratory workups were unremarkable except for severe thrombocytopenia. Brain computed tomography (CT) scan was normal. Fundoscopy revealed bilateral papilledema; thus, magnetic resonance venography (MRV) was performed and demonstrated occlusion of the sagittal, supra-tentorial, and left transverse occipital sinuses that are consistent with CVT (Figure 1).

On admission, levetiracetam 500 mg bid was started. We could not start the optimal treatment with warfarin because of the high risk of intracranial bleeding; thus, enoxaparin (1 mg/kg) was administrated. Unfortunately, IVIG is not available in our country; therefore, the only emergent treatment for thrombocytopenia was platelet transfusion and high-dose methylprednisolone (1 mg/kg). In the following days, the patient's state improved significantly. On the 7^th^ day, the patient's platelets stabilized above 100 × 10^9^/L and her neurological symptoms improved obviously; thus, warfarin was initiated instead of enoxaparin with close monitoring. On the 10^th^ day, she was neurologically intact without any abnormal neurologic signs or symptoms and the patient has discharged on levetiracetam 500 mg per day and prednisolone 40 mg per day. On clinical and laboratory follow-up, she had no complaints. However, her thrombocytopenia has recurred. Unfortunately, CD20 inhibitors and thrombopoietin receptors agonists are unavailable in our country. Therefore, splenectomy was performed. Her postoperative follow-up for 6 months was normal and her platelet count was stabilized above 150 × 10^9^/L.

## 3. Discussion

ITP is an autoimmune disorder characterized by a low platelet count below the level of 100 × 10^9^/L. This reduction in the platelet level is attributed to its destruction mediated by autoimmune antibodies [2, 3].

The spectrum of clinical manifestations is very widened to include completely asymptomatic cases as well as life-threatening bleeding. Some patients may describe no symptoms and their condition may be diagnosed accidentally. However, in other settings, patients may express bleeding manifestations such as purpura, epistaxis, petechiae, and bruising, as our patient. In some rare cases, a lethal hemorrhage may occur especially intracranial or gastrointestinal bleeding [2].

Although bleeding is the main manifestation of ITP, the paradoxical process, that is, thrombosis, can also occur. Furthermore, some previous studies suggested that patients with ITP have an increased risk for thrombosis [1–4].

Many hypotheses can explain the thrombotic events in patients with ITP, and two of them are associated with the disease etiology, the elevated level of antiphospholipid antibodies and platelet microparticles in patients with ITP. The other suggestions are associated with the treatment, as almost all of the agents used in the management of ITP play a role in the occurrence of the thrombotic phenomenon. Exposure to IVIG or thrombopoietin receptor agonist increases the risk of thrombosis. However, the corticosteroids may also contribute to the thrombotic process by causing a hypercoagulable state, thus leading to thrombosis. In addition, some patients develop a thrombotic event just after splenectomy that can be explained by the rebound elevation of platelet level. Taking into account other risk factors (such as age, obesity, smoking, hypertension, diabetes mellitus, dyslipidemia, splenectomy, and oral contraceptive agents), the presence of ITP or its treatment should be considered as a risk factor for thromboembolic events [1–5].

According to the European Stroke Organization guidelines, it is recommended preferentially to use low molecular weight heparin in the acute phase rather than direct oral anticoagulants. The combination between ITP (a major bleeding risk factor) and CVT (a thromboembolic event) has carried a major challenge to the treatment of our patient.

We herein present a case of a young female who was diagnosed with ITP. After the exclusion of any other secondary causes, the diagnosis of ITP was established and the patient started treatment with prednisolone 1 mg/kg per day. On follow-up, the patient demonstrated fluctuating response, as the platelet level wavered and was not increasing steadily. However, our patient continued the treatment with corticosteroids with a close observation of any bleeding manifestations and did recurrent laboratory tests. In November 2020, the patient presented with a severe headache, altered consciousness, and a generalized seizure. According to her past medical history, we expected intracranial bleeding causing neurological manifestations. However, the MRV imaging revealed cerebral venous thrombosis (Figure 1). The venous thrombosis was an unexpected finding in a patient whose condition tends to be bleeding. Furthermore, her platelet level at the time of the neurological manifestations was 5 × 10^9^/L that made the case more complicated. The cause of her venous thrombosis remains unclear as the patient is young and has no other risk factors. We believe that the combination of ITP and ITP treatment with corticosteroids have both contributed to a hypercoagulable state that was complicated as CVT. The patient was treated with enoxaparin 1 mg/kg subcutaneous daily, which was administrated cautiously with direct and close observation of any bleeding demonstrations as her platelet level was below the normal limits. In the following days, the neurological symptoms diminished and the patient's state improved obviously. Based on the poor response for treatment with corticosteroids for the ITP, the patient went ultimately through splenectomy and the long-term follow-up demonstrated a good response with a remarkable increase in the level of platelets.

In conclusion, we believe that ITP and ITP treatment methods should be taken into consideration as risk factors for thromboembolic events.

## Data Availability

No data were used to support this study.

## Abbreviations

ITP: Immune thrombocytopenic purpura
IVIG: Intravenous immunoglobulin
CVT: Cerebral venous thrombosis
ICP: Increased intracranial pressure
CT: Computed tomography
MRV: Magnetic resonance venography.
